# The complete mitochondrial genome of the photosymbiotic sea slug *Berghia stephanieae* (Valdés, 2005) (Gastropoda, Nudibranchia)

**DOI:** 10.1080/23802359.2021.1914211

**Published:** 2021-07-12

**Authors:** Jenny Melo Clavijo, Franziska Drews, Marcello Pirritano, Martin Simon, Abdulrahman Salhab, Alexander Donath, Silja Frankenbach, João Serôdio, Sabrina Bleidißel, Angelika Preisfeld, Gregor Christa

**Affiliations:** a Bergische Universität Wuppertal, Fakultät für Mathematik und Naturwissenschaften, Zoologie und Biologiedidaktik, Wuppertal, Germany; b Bergische Universität Wuppertal, Fakultät für Mathematik und Naturwissenschaften, Chemie und Biologie, Molekulare Zellbiologie und Mikrobiologie, Wuppertal, Germany; c Department of Genetics, Saarland University, Saarbrucken, Germany; d Zoologisches Forschungsinstitut und Museum Alexander Koenig, Bonn, Germany; e Department of Biology and CESAM, Centre for Environmental and Marine Studies, University of Aveiro, Aveiro, Portugal

**Keywords:** Nudibranchia, Aeolidiidae, Nudipleura, Photosymbiosis, Mollusca

## Abstract

*Berghia stephanieae* (Nudibranchia, Cladobranchia) is a photosymbiotic sea slug that feeds exclusively on sea anemones from the genus *Exaiptasia*. It then specifically incorporates dinoflagellates belonging to the Symbiodiniaceae obtained from their prey. Here, we present the complete mitochondrial genome sequence of *B. stephanieae* combining Oxford Nanopore long read and Illumina short-read sequencing data. The mitochondrial genome has a total length of 14,786 bp, it contains the 13 protein-encoding genes, 23 tRNAs, and two rRNAs and is similar to other nudibranchs except for the presence of a duplicated *tRNA-Ser 1.*

The aeolid nudibranch *Berghia stephanieae* (Valdés 2005) (Nudibranchia, Cladobranchia) is a stenophagous species that preferentially preys on the photosymbiotic sea anemone *Exaiptasia diaphana* (Rapp 1829). The slug digests all the anemones’ tissue and incorporates the dinoflagellate symbionts Symbiodiniaceae Fensome et al. 1993, in epithelial cells of the digestive gland system (Valdés 2005; Carmona et al. 2014). Once ingested, the symbionts are retained photosynthetically active for about 10 days (Mies et al. 2017), but the slugs are even able to overcome prolonged starvation periods up to 48 days apo-symbiotically (symbiont-free) (Bleidissel 2010). Further, because apo-symbiotic adults lose their biomass in the same manner as photosymbiotic ones, the photosymbiotic relationship of *B. stephanieae* and Symbiodiniaceae is rather considered as non-mutalistic than a stable one (Mies et al. 2017; Monteiro et al. 2019). Nevertheless, *B. stephanieae* is an important species to understand the evolution of photosymbiosis in Cladobranchia, because the species seems to be in a transitional state between non-photosymbiotic and photosymbiotic. To better understand the genomic adaptations needed to evolve a stable photosymbiosis (Melo Clavijo et al. 2018), the metabolism of the mitochondrial genome can give valuable insights into a potential connectivity of the host and the symbiont (Rauch et al. 2017). As a first step toward more comprehensive studies, we sequenced the mitochondrial genome of *B. stephanieae* using a combination of Oxford Nanopore long-read and Illumina short-read sequencing.

Specimens of *B. stephanieae* were purchased from a local provider (Seepferdchen24 Meeresaquaristik GmbH, Posthausen) in February 2019 and cultivated in our lab at 25 °C, at a day/night cycle of 12 h/12h. Voucher material was preserved in 96% ethanol and stored in the Biobank at the Zoological Research Museum Alexander Koenig (Bonn, Germany, voucher no. ZFMK-TIS-53240, biobank@leibniz-zfmk.de). Seven specimens of *B. stephanieae* were frozen in liquid nitrogen and total DNA was extracted using a modified protocol based on the E.Z.N.A^®^Mollusc DNA Kit, Omega (Georgia, USA) and after Schalamun et al. (2019) (Supplementary material S1). The genomic library preparation was performed using the 1 D Ligation Sequencing Kit SQK-LSK109, Oxford Nanopore Technologies (Oxford, UK) for long-read sequencing on a MinION device, using a modified manufacturer’s protocol (Supplementary material S1) generating about 13 GB of long reads. An additional library (insert size 100 bp, single end) was prepared using the Nextera DNA Library Prep Kit (California, USA) for Illumina sequencing on a HiSeq2500 platform resulting in approximately 5.5 GB of data. A hybrid assembly was done using SPAdes V3.14.1 (Nurk et al. 2013; Antipov et al. 2016), the assembled genome was annotated using the MITOS2 webserver (Bernt et al. 2013; Donath et al. 2019), and annotations were manually edited using Geneious 9.1.5. (https://www.geneious.com). Duplicated tRNAs were further confirmed with ARWEN v.1.2 (Laslett and Canback 2008).

The mitochondrial genome of *B. stephanieae* (GenBank accession number: MW027646) has a total length of 14,786 bp and consists of 13 protein-coding genes, two ribosomal RNA (rRNA) genes, and 23 tRNA genes. The base composition of the mitogenome is 26% A, 15% C, 21% G, and 38% T. The gene order is as follows: *tRNA-Lys (aaa), cox1, tRNA-Val (gta),* the large-subunit rRNA *(rrnL), tRNA-Leu (cta) 1, tRNA-Ala (gca), tRNA-Pro (cca), nad6, nad5, nad1, tRNA-Tyr (tac), tRNA-Trp (tga), nad4L, cob, tRNA-Asp (gac), tRNA-Phe (ttc), cox2, tRNA-Gly (gga), tRNA-His (cac), tRNA-Cys (tgc), -tRNA-Gln (caa), -tRNA-Leu (tta) 2, -atp8, -tRNA-Asn (aac), -atp6, -tRNA-Arg (cga), -tRNA-Glu (gaa), -*the small-subunit rRNA *(rrnS), -tRNA-Met (atg), -nad3, -tRNA-Ser (tca) 2, tRNA-Ser (agc) 1, tRNA-Ser (aga) 1, nad4, -tRNA-Thr (aca), -cox3, tRNA-Ile (atc), nad2.* The mitogenome of *B. stephanieae* is similar in size, base composition, has the same coding regions and gene arrangement compared to all publicly available nudibranch mitochondrial genomes (Sevigny et al. 2015; Karagozlu, Sung, Lee, Kim, et al. 2016; Karagozlu, Sung, Lee, Kwak, et al. 2016; Xiang, Lin, Wang, et al. 2016; Xiang, Lin, Zhao, et al. 2016; Lin et al. 2017; Yu et al. 2018; Dinh Do, Choi, et al. 2019; Dinh Do, Kim, et al. 2019) and only differs in the presence of a duplicated *tRNA-Ser 1*.

Full-length mitochondrial genome sequences of 20 Nudipleura species were downloaded from NCBI and aligned using MAFFT (Auto mode) V7.222 (Katoh and Stanley 2013). A phylogenetic tree was built based on the maximum likelihood criterion using IQ-TREE version 2.0.5 (Minh et al. 2020) with the Model Finder Plus option (-m TEST), 1000 bootstrap replicates, and *Aplysia californica* J. G. Cooper 1863 set as outgroup. *Berghia stephanieae* clustered with the other Cladobranchia species, and forms a monophyletic clade with *Sakuraeolis japonica* (Baba 1937) and *Hermissenda emurai* (Baba 1937), that corresponds to the superfamily Aeolidioidea (Figure 1).

**Figure 1.**
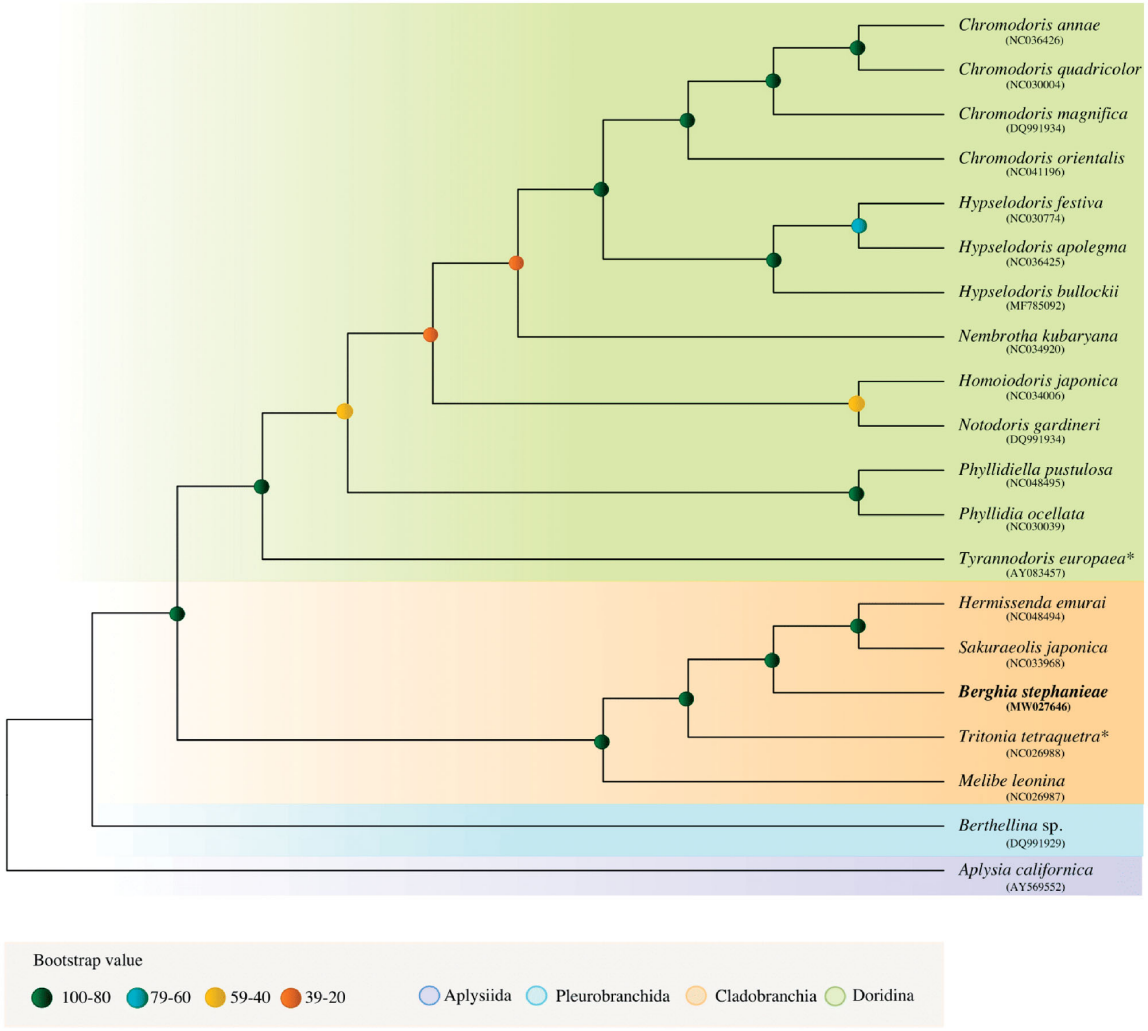
The molecular phylogeny of *Berghia stephanieae* and other nudibranchs based on the whole mitochondrial genome. The phylogenetic tree was calculated under the maximum-likelihood optimality criterion and 1,000 bootstrap replicates using *Aplysia californica* as outgroup. The accepted names (WoRMS Editorial Board, 2020) for *Tyrannodoris europaea* (synonym *Roboastra europaea*) and *Tritonia tetraquetra* (synonym *Tritonia diomedea*) were used (*).

## Supplementary Material

Supplemental Material

## Data Availability

The data that support the findings of this study are openly available in Figshare (www.figshare.com) at http://doi.org/10.6084/m9.figshare.12994064

## Data accessibility statement

The mitogenome sequences of *Chromodoris annae* Bergh 1877 (NC_036426) (Lin et al. 2017), *Chromodoris quadricolor* (Rüppell and Leuckart 1830) (NC_030004) (Xiang, Lin, Zhao, et al. 2016), *Chromodoris magnifica* (Quoy and Gaimard 1832) (DQ991931) (Medina et al. 2011)*, Chromodoris orientalis* Rudman 1983 (NC_041196) (Yu et al. 2018), *Hypselodoris festiva* (Adams 1861) (NC_030774) (Karagozlu, Sung, Lee, Kwak, et al. 2016), *Hypselodoris apolegma* (Yonow 2001) (NC_036425) (Lin et al. 2019), *Hypselodoris bullockii* (Collingwood 1881) (MF785092) (Lin et al. 2019), *Nembrotha kubaryana* Bergh 1877 (NC_034920) (Xiang et al. 2017), *Homoiodoris japonica* Bergh 1882 (NC_034006) (Liu et al. 2016), *Notodoris gardineri* Eliot 1906 (DQ991934) (Medina et al. 2011), *Phyllidiella pustulosa* (Cuvier 1804) (NC_048495) (Dinh Do, Choi, et al. 2019), *Phyllidia ocellata* Cuvier 1804 (NC_030039) (Xiang, Lin, Wang, et al. 2016), *Tyrannodoris europaea* (García-Gómez 1985) (registered as its synonym *Roboastra europaea* García-Gómez 1985 AY083457) (Grande et al. 2002), *Hermissenda emurai* (NC_048494) (Dinh Do, Kim, et al. 2019), *Sakuraeolis japonica* (NC_033968) (Karagozlu, Sung, Lee, Kim, et al. 2016)*, Tritonia tetraquetra* (Pallas 1788) (registered as its synonym *Tritonia diomedea* Bergh 1894 NC_026988) (Sevigny et al. 2015), *Melibe leonina* (Gould 1852) (NC_026987) (Sevigny et al. 2015), *Berthellina* sp. Gardiner 1936 TLT-2006 (DQ991929) (Medina et al. 2011), and *Aplysia californica* (AY569552) (Knudsen et al. 2006) are accessible via download from GenBank. Mitogenome data supporting this study are openly available in GenBank at: https://www.ncbi.nlm.nih.gov/nuccore/MW027646. Associated BioProject, SRA, and BioSample accession numbers are https://www.ncbi.nlm.nih.gov/bioproject/PRJNA684024, SRR13242883, SRR13242884, SRR13242885, and SAMN17050363, respectively.
