# Supplementary material for: The complete mitochondrial genome of the photosymbiotic sea slug Berghia stephanieae (Valdés, 2005) (Gastropoda, Nudibranchia)
Source: Mitochondrial DNA B Resour. 2021 Jul 12;6(8):2281–4. doi: 10.1080/23802359.2021.1914211 (PMC8279152; doi:10.1080/23802359.2021.1914211)
Supplement: Supplemental Material [file TMDN_A_1914211_SM6816.docx]

**Appendix 1. DNA extraction protocol and library preparation for nanopore sequencing**

Seven specimens of *Berghia stephanieae*, 17 mg each, were frozen in liquid nitrogen. The tissue was homogenized using 350 µl of the lysis buffer ML1 from the E.Z.N.A^®^ Mollusc DNA Kit, Omega (Georgia, USA) and 25 µl of Proteinase K and heated to 60 °C for 1 h. The samples were then cleaned adding 350 µl of chloroform: isoamyl (24:1) and the phases were separate by centrifugation for 5 min at 8000 g at room temperature. The aqueous phase was collected and transferred to a new tube. One volume of buffer BL of the E.Z.N.A^®^ Mollusc DNA Kit and 10 µl RNase A/T (Thermo Scientific, Massachusetts, USA) were added and incubated at 70 °C for 10 min. A second cleaning step was done by adding one volume of chloroform: isoamyl (24:1) to the samples and centrifuged them at 8000 g at room temperature and the aqueous phase recovered. The DNA in the aqueous phase was precipitated adding 0.1 volume 3M sodium acetate, and 1 ml 96% ethanol, and further centrifuged for 5 min at 5000 g at room temperature. The supernatant was discarded and the pellet was washed with 1 ml 70% ethanol and centrifuged for 10 min at 8000 g at room temperature. After a second washing step with 1 ml 70% ethanol, the pellet was air dried and eluted overnight at room temperature in 100 µl of Elution Buffer E.Z.N.A^®^ Mollusc DNA Kit. The concentration and purity of the isolated total DNA was measured with Nanodrop and Qubit, respectively.

Library preparation for long-read sequencing with the MinION (Oxford Nanopore, Oxford, UK) was performed using the 1D SQK-LSK109 ligation kit, following the manufacturers’ recommendations. Briefly, 2 µg of pre-warmed (50 °C) isolated genomic DNA were end-repaired and prepared using the NEBNext FFPE DNA repair Mix (New England Biolabs, Massachusetts, USA) and NEBNext End repair/dA-tailing Module (New England Biolabs, Massachusetts, USA) for one hour at 20 °C followed by one hour at 65 °C. The sample was then incubated for 15 min at room temperature with AMPure XP beads (1:1, v/v) (Beckman Coulter, California, USA) previously acclimated at room temperature for 30 min. The sample was then briefly spinned down using a mini centrifuge (LMS, Tokyo, Japan), and the tube subsequently placed on a magnetic rack allowing the beads to concentrate at the side of the magnet for 5 min. Afterwards, the supernatant was removed and the beads washed by adding 500 µl of freshly prepared 70% ethanol. The washing step was repeated twice. The cleaned pellet was then eluted in 62 µl of nuclease-free water at 37 °C for 30 min. Subsequently, the concentration and purity of the DNA was checked using a Nanodrop and only proceeded if the A260/A280 ratio was 1.8. Then, 25 µl of LNB, 10 µl of NEBNext Quick T4 DNA Ligase, and 5 µl of the Adapter Mix AMX were added to the DNA and incubated at room temperature for 1 h. Next, 40 µl of AMPure XP beads were added and mixed by inversion for 15 min at room temperature, and placed on the magnetic rack for 5 min. The supernatant was discarded and 250 µl of L Fragment Buffer LFB were added to the beads mix in order to select fragments longer than 3 kbp. This washing step was repeated once. After removing all the remaining LFB buffer from the second washing step, the pellet was resuspended in 15 µl Elution Buffer (pre-warmed at 50 °C) and incubated at 37 °C for 20 min. For sequencing, 15 µl of final DNA were mixed with 36 µl of sequencing buffer SQB, and 24 µl of loading beads LB, mixed immediately before use, having in total 75 μl DNA in solution to be loaded onto the flow cell. The flow cell was primed with a priming solution made out of 30 μl Flush Tether FLT directly mixed with 1 ml of Flush Buffer FLB. First, 800 μl of the priming solution were added into the sample port with a closed SpotON port. After 5 min the remaining 200 μl of the priming solution were added, but with an open SpotON port. Next, the prepared DNA library was loaded drop-by-drop into the open SpotON port. The sequencing run was monitored on a Ubuntu 18.04. platform using MINKNOW v.2.2. basecalling was performed using Guppy v.3.0.3.
